# Irrigation Optimization via Crop Water Use in Saline Coastal Areas—A Field Data Analysis in China’s Yellow River Delta

**DOI:** 10.3390/plants12101990

**Published:** 2023-05-15

**Authors:** Jing Li, Deyao Liu, Yitao Zhang, Zhen Liu, Lingqing Wang, Huarui Gong, Yan Xu, Shanqing Lei, Hanyou Xie, Andrew Binley

**Affiliations:** 1 Key Laboratory of Ecosystem Network Observation and Modeling, Institute of Geographic Sciences and Natural Resources Research, Chinese Academy of Sciences, Beijing 100101, China; liudeyao20@mails.ucas.ac.cn (D.L.); zhangyt@igsnrr.ac.cn (Y.Z.); wanglq@igsnrr.ac.cn (L.W.); xuy.17s@igsnrr.ac.cn (Y.X.); leishanqing22@mails.ucas.ac.cn (S.L.); 2 College of Resource and Environment, University of Chinese Academy of Sciences, Beijing 100049, China; 3 Yellow River Delta Modern Agricultural Engineering Laboratory, Institute of Geographic Sciences and Natural Resources Research, Chinese Academy of Sciences, Beijing 100101, China; liuzhen@igsnrr.ac.cn (Z.L.); hrgong@igsnrr.ac.cn (H.G.); 4 Key Laboratory of Plant Nutrition and Fertilizer, Ministry of Agriculture and Rural Affairs/Institute of Agricultural Resources and Regional Planning, Chinese Academy of Agricultural Sciences, Beijing 100081, China; 5 Lancaster Environment Centre, Lancaster University, Lancaster LA1 4YQ, UK; a.binley@lancaster.ac.uk

**Keywords:** saline coastal farmland, crop rotation system, salt stress, water budget, crop coefficient, water use efficiency

## Abstract

Freshwater resources are becoming increasingly scarce in coastal areas, limiting crop productivity in coastal farmlands. Although the characteristic of crop water use is an important factor for water conservation in coastal farmlands, it has not been studied extensively. This study aimed to depict the water use process of soil–plant systems under saline stress in coastal ecosystems and optimize water management. An intensive observation experiment was performed within China’s Yellow River Delta to identify the water use processes and crop coefficients (K_C_) and also quantify the impacts of salt stress on crop water use. The results show that shallow groundwater did not contribute to soil water in the whole rotation; K_C_ values for wheat–maize, wheat–sorghum, and wheat–soybean rotation systems were 45.0, 58.4, and 57% less, respectively, than the FAO values. The water use efficiency of the maize (8.70) and sorghum (9.00) in coastal farmlands was higher than that of the soybean (4.37). By identifying the critical periods of water and salt stress, this paper provides suggestions for water-saving and salinity control in coastal farmlands. Our findings can inform the sustainable development of coastal farmlands and provide new insights to cope with aspects of the global food crisis.

## 1. Introduction

With climate change and population growth, global food security is increasingly challenged. Coastal agricultural ecosystems support 40% of the world’s population [1], but are affected by seawater intrusion, soil degradation, and salinization, all of which limit the productivity and sustainability of these ecosystems [2,3]. In saline coastal soils, soil water is important not only for crop growth but also for soil salt regulation. The accurate evaluation of crop water use (CWU) characteristics under saline coastal soil conditions is important for taking appropriate agricultural management measures to improve the productivity of coastal agricultural ecosystems and global food security.

Analyzing CWU processes, including crop evapotranspiration (ET_C_), in coastal farmlands is a critical first step toward designing irrigation schemes that can fully deal with saline stress [4,5]. With saline stress, large amounts of Na^+^ and Cl^−^ plasma accumulate in the root zone. This increases soil solute potential and physiological water shortage in crops, thereby limiting CWU [6,7]. On the other hand, groundwater levels are typically relatively shallow in saline coastal areas. Changes in the water level result in changes to the total available soil water in the root zone, which in turn affect CWU [8]. As these factors affect CWU in saline farmlands, it is necessary that they are accurately analyzed and quantified.

The conventional approach to estimating ET_C_ in saline coastal farmlands is subject to many limitations and uncertainties. The observation methods at the farm scale include the lysimeter method [9,10], the Bowen ratio method [11,12], the eddy covariance method [13,14], etc. However, because soil salinity in coastal farmlands leads to erosion and degradation of the sensors and other parts of monitoring equipment, the accuracy and reliability of the data collected can be limited. Remote sensing has also been applied in such areas; however, such an approach is often not suitable at the farmland scale because of the resolution of the images [15,16]. The K_C_-ET_O_ approach can be used to measure local ET_C_ via the product of the crop coefficient (K_C_) and reference evapotranspiration (ET_O_) [17], which is widely used at the farmland scale [18,19,20]. There are studies where the K_C_-ET_O_ approach has been used to estimate ET_C_ of some halophytes [21], potatoes, and broad beans [22] in saline coastal soils and to quantify local Kc. Such studies illustrate the applicability of this approach in saline coastal farmlands.

However, the lack of Kc values for staple food crops in saline coastal farmlands results in considerable uncertainties in the evaluation of ET_C._ This makes it impossible to accurately identify CWU and to design optimal crop water management schemes in saline coastal farmlands. In addition, the exchange of water between the plant root zone and groundwater (deep percolation or capillary rise) in coastal areas is often a complex process [23] and an important part of soil moisture and, therefore, the accurate quantification of regional ET_C_.

Numerical models are widely used to simulate the exchange of fluids in the surface environment. The HYRUS-1D model is ideal for analyzing flux at the base of saline coastal soil since the model is well established and underpinned by physically based parameters, which are accessible from field observations [24,25,26]. It can therefore be used to evaluate ET_C_ by determining K_C_ in saline coastal farmlands via in situ observations. This can ultimately lead to the accurate evaluation of CWU of staple food crops in saline coastal farmlands.

Previous studies show that developing reasonable crop rotation systems based on CWU characteristics is critical in the effort to improve crop productivity [27,28]. The Chinese Yellow River Delta (YRD) is located at the estuary of the lower reach of Yellow River that is close to the Bohai Sea, and it is a typical saline coastal soil area. The dominant crop rotation system in saline coastal farmlands in the YRD is winter wheat–summer maize. There is also a small area under a winter wheat–soybean or winter wheat–sorghum rotation system. Due, however, to the combined effect of salt and water stress, farmland productivity in the region is limited. It is therefore important to adjust the crop rotation system for the highest crop productivity in the YRD. To determine alternative crop rotation systems and design farmland management measures to meet this high productivity, an accurate evaluation of CWU characteristics in the region is required.

In this study, three crop rotation systems (wheat–maize, wheat–soybean, and wheat–sorghum) were analyzed for water use process in the YRD. The objectives of the study were to determine (i) the relationship between crop root zone and shallow groundwater by identifying the main soil water loss and soil water supply factors in the study area, (ii) the K_C_ of the main crops in saline coastal farmland so as to accurately evaluate ET_C_ of the different crop rotation systems, and (iii) the critical stage of crop water shortage and water use efficiency of the different crop rotation systems.

## 2. Results

### 2.1. Spatio-Temporal Variations in Soil Water

**Figure 1:**
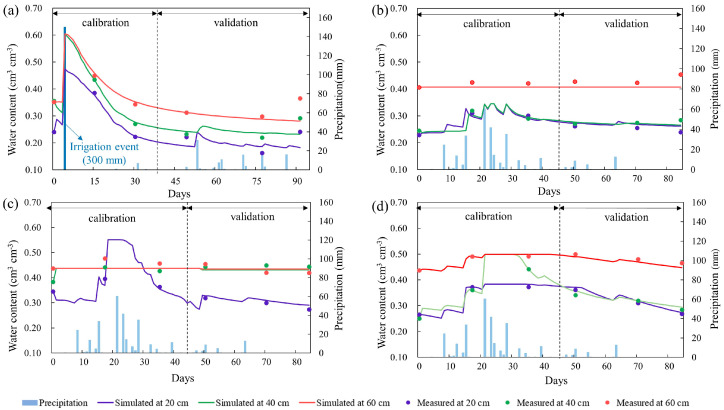
Simulated (lines) and measured (points) values of soil water content in (**a**) a wheat field, (**b**) a sorghum field, (**c**) a maize field, and (**d**) a soybean field at three soil observation depths (20, 40, and 60 cm soil depth) in the Yellow River Delta study area.

The simulations for the four crops had, overall, a good fit to the observed data during the calibration and validation stages (Table S1). For the calibration stage, the performances of the four crops were satisfactory (*R*^2^ ≥ 0.88; RMSE ≤ 0.022; NSE ≥ 0.82). However, the results of the maize calibration (*R*^2^ = 0.88) were worse than those for the other three crops (*R*^2^ = 0.97, 0.98, 0.99). This is because the model underestimated the soil water content in the 60 cm soil depth in the maize field (Figure 1c), but the model results for maize were considered reliable as the subsequent validation had a good fit (*R*^2^ = 0.98). The validations for soybean (*R*^2^ = 0.99) and sorghum (*R*^2^ = 0.92) were also good. However, the model simulations for wheat were relatively worse (*R*^2^ = 0.60), which was mainly due to the model underestimating the increase in soil moisture in the last few days of the studied period (Figure 1a). In general, the model of soil water transport in the saline coastal farmland study area of the YRD was considered satisfactory for the three crop fields. 

The water content in the topsoil (0–20 cm) of maize, soybean, and sorghum fields changed rapidly in summer. However, the water content in the topsoil in the wheat field was relatively stable in spring (Figure 1). Similarly, significant differences were noted in the water content in the deep soil (20–60 cm) between the winter and summer crops. The change in water content in the deep soil was relatively consistent with that in the topsoil layers in the wheat fields. However, for summer crops, the change in water content in the deep soil was relatively stable over time, whereas variation was seen in the topsoil layers.

### 2.2. Water Budget and Groundwater Effect

**Figure 2:**
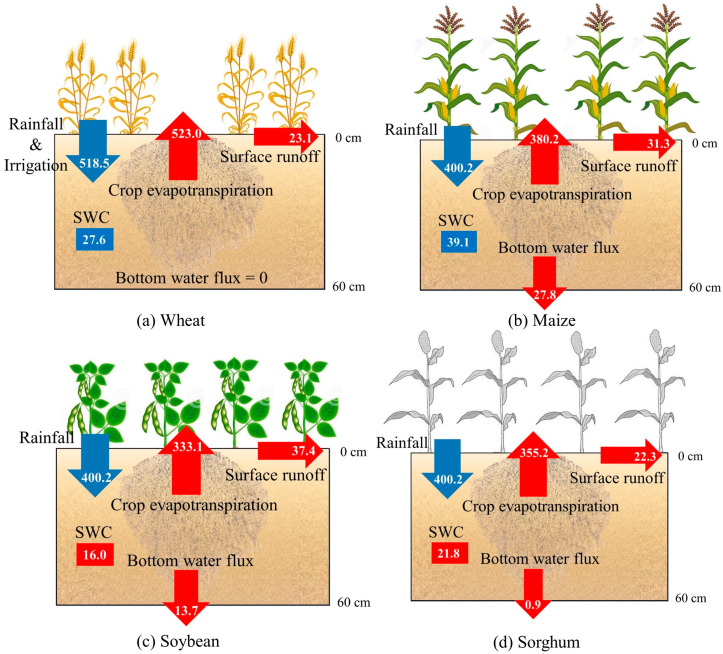
Water budget for the wheat, maize, soybean, and sorghum in the Yellow River Delta study area. Note that SWC is change in soil water content [mm]; red denotes water loss, and blue denotes water gain. Rainfall, irrigation, and surface runoff were obtained by in-site observations; evapotranspiration was obtained by the Penman–Monteith equation based on climate data; bottom water flux was obtained by the simulations from HYDUS-1D model; and SWC was then calculated by water balance equation.

Based on the measured data and water balance equation, the water budget was quantified for the four crops (Figure 2) and three rotation systems (Figure S2). Surface runoff was low in the wheat field (23.1 mm), accounting for 4.42% of the CWU. As summer progressed, precipitation increased, and surface runoff gradually increased. Surface runoff in the soybean, maize, and sorghum fields was 37.4, 31.3, and 22.3 mm, respectively, accounting for 11.20, 8.23, and 6.28% of the total water use. For the one-year rotation in the YRD, surface runoff was highest in the wheat–sorghum field (60.5 mm), followed by the wheat–maize field (54.4 mm) and then the wheat–soybean field (45.4 mm).

For the entire crop growth period, the flow of water through the root zone to groundwater was complex, affected by factors such as groundwater fluctuation and climate. Here only the overall cumulative water flux from the base of the bottom soil layer for the whole growth period was considered and characterized as DP for positive flux and CR for negative flux. DP varied considerably (0–27.8 mm) for the growth periods of the different crops. The bottom flux was 0.0 mm for the wheat growth period, meaning that DP was offset by CR. In summer, DP occurred in the simulations of every crop field; it was highest for the maize field (27.8 mm), followed by the soybean field (13.7 mm) and the sorghum field (0.9 mm), accounting for 7.32, 4.11, and 0.25%, respectively, of the water use of each crop. For the one-year rotation in the YRD, water use in the wheat–maize rotation was the largest (903.2 mm), followed by wheat–sorghum (878.2 mm) and the wheat–soybean rotation (856.1 mm).

### 2.3. Crop Coefficient Determination

**Figure 3:**
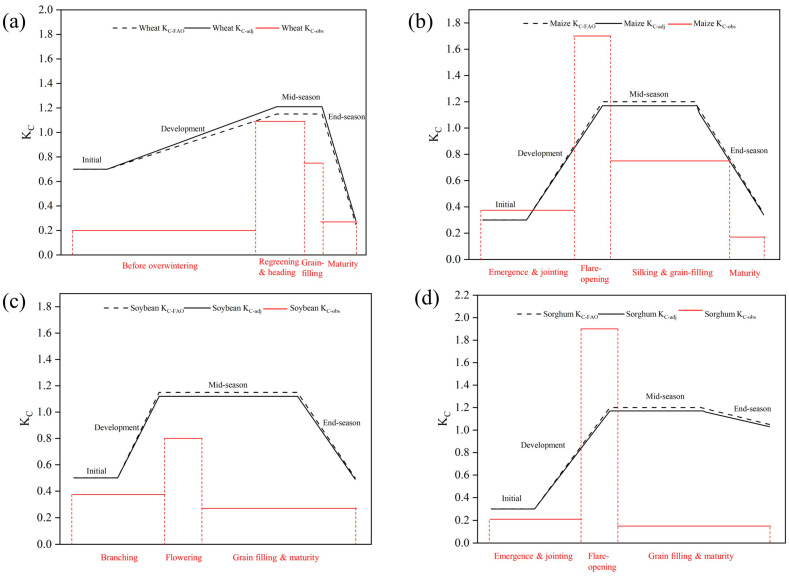
Comparison of K_C-FAO,_ K_C-adj_, and K_C-obs_ for different growth stages of (**a**) wheat, (**b**) maize, (**c**) soybean, and (**d**) sorghum in the Yellow River Delta study area.

The local spring flood and summer rainfed irrigation scheme led to varying degrees of water stress in the three crop rotation systems (Figure 3). For wheat, K_C-obs_ for the whole growth period was lower than the ideal value. If K_C-adj_ values were adopted in the YRD, the local K_C_ value of wheat prior to the overwintering (0.20), regreening and heading (1.09), grain-filling (0.75), and maturity (0.27) stages would be overestimated by 77.0, 9.0, 38.0, and 57.0%, respectively. The same trend of results was also observed for soybean. The local K_C_ values for soybean at branching (0.37), flowering (0.8), and grain-filling and maturity (0.27) were overestimated by 39.2, 31.4, and 47.9%, respectively, compared with the K_C-adj_ values. For sorghum and maize, the flare-opening stage was the key growth stage for the whole growth period for which K_C-obs_ was much higher than K_C-adj_. The K_C-adj_ values for maize and sorghum in this period were underestimated by 39.0 and 45.0%, respectively. For the entire growth period, however, the local K_C_ values for maize and sorghum were low on the whole. If the K_C-adj_ values were adopted under the spring flood and summer rainfed irrigation scheme in the YRD, the local average K_C_ values for the wheat–maize, wheat–sorghum, and wheat–soybean rotation systems would be overestimated by 45.0, 58.4, and 57%, respectively.

### 2.4. Crop Water Use Efficiency and Deficit

**Figure 4:**
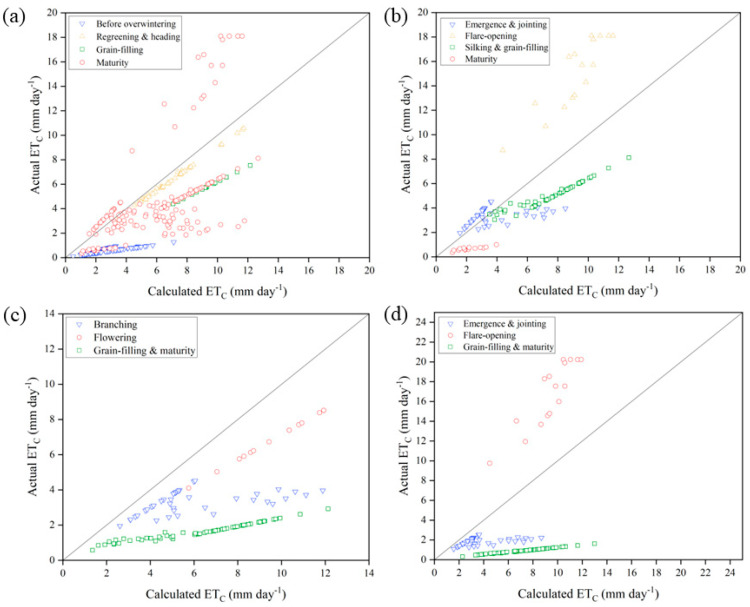
Comparison of daily actual ET_C_ (ET_C-obs_) and calculated ET_C_ (ET_C-adj_) in different growth stages of (**a**) wheat, (**b**) maize, (**c**) soybean, and (**d**) sorghum in the Yellow River Delta study area.

The actual and calculated daily water use in the YRD study area was obtained using K_c_×ET_O_ (Figure 4). There was an obvious water deficit in the crop fields over the observed period. For soybean (Figure 4c), water demand was not met for the whole growth period. The average water use in each growth stage was only 54.8, 71.4, and 25.9% of that under ideal conditions. For wheat (Figure 4a), water use was the lowest prior to overwintering (0.54 mm day^−1^). With spring irrigation, available water in the next two growth stages increased, and daily used water increased accordingly (6.27 and 5.77 mm day^−1^, respectively). Although the average daily water use for wheat varied considerably at maturity stage, the water use was still limited for most of the time (2.55 mm day^−1^). The water use by maize (Figure 4b) was similar to that by sorghum (Figure 4d), with the highest CWU in the flare-opening stage (14.96 and 16.72 mm day^−1^, respectively). The difference was that water stress in the other two stages of maize was small, except for the maturity stage (0.67 mm day^−1^), which is not far from the ideal value. In addition, the other growth stages of sorghum showed large water stress. The water use during the silking and grain-filling stages was 0.92 mm day^−1^, only 12.7% of the ideal value.

Further, the salt stress factor K_S_ was calculated for each crop at the various growth stages (Table 1). The results show that on the whole, only the flare-opening stages of sorghum and maize did not show salt stress, with K_S_ values of 1.651 and 1.845, respectively. Concurrently, however, the K_S_ value for soybean at flowering stage was only 0.714. All the other growth stages of soybean showed different degrees of salt stress (0.132–0.912). All the crops showed severe salt stress in the early and late growth stages, which slightly lessened in the middle growth stages. Using the early growth stage as the reference point, wheat showed the highest salt stress (0.233), followed by sorghum (0.550), soybean (0.608), and then maize (0.980). Sorghum suffered the worst salt stress (0.132) at the grain-filling and maturity stages.

Water use efficiency (WUE) was calculated from the ratio of grain yield (GY) to ET_C-obs_ for the whole rotation (Table 1). The WUE of soybean was lowest (4.37) because of the combined effect of salt and water stress. The WUEs of maize (8.70) and sorghum (9.00) were not very different. On the whole, WUE of the three crop rotation systems was in the order of wheat–sorghum (8.11) > wheat–maize (8.00) > wheat–soybean (6.28).

## 3. Discussion

### 3.1. Soil Water Supply and Loss

Due to the precipitation intensification in summer, infiltration from precipitation led to significant changes in the water content within the topsoil (0–20 cm) under summer crops. The change in water content of the topsoil under spring wheat was mainly driven by irrigation (300 mm). After irrigation, the soil water content continued to decline due to evapotranspiration losses. As water supply as rainfall was small at that time, the overall change in soil water content was relatively small. Similarly, there were significant differences in water content of the deep soil (20–60 cm) between the two cropping seasons. The change in deep soil and topsoil water in wheat field was consistent, affected by irrigation and insensitive to precipitation. In summer crop fields, water content of the deep soil was stable as it was not affected by precipitation and evapotranspiration. Studies in shallow groundwater regions also show stable deep soil water content during the season of crop growth [29,30].

Studies have also shown that in areas with potential evapotranspiration greater than average precipitation, CR and DP are difficult to estimate due to the effect of climatic, soil, crop growth, and agronomic conditions [31,32]. The water flux from the bottom soil in the wheat field was 0 mm for the whole growth period, implying that CR offset DP. Because of the long period of low temperature and rainfall in the early stage of wheat growth and significant increase in rainfall, temperature, and groundwater level in the later stage, neither DP nor CR occurred in the bottom soil layer, which is much less than values reported in other studies. For example, in the semi-arid region of India, DP in the summer soybean field equal to 159.1 mm has been reported [33]. In the North China Plain with a deep water table, DP in summer maize field has been observed to account for 30% of the total annual water supply [34]. Studies have also shown that groundwater can account for over 40% (200 mm) of the evapotranspiration of summer maize in river zones with shallow groundwater [35]. This was different for the saline coastal farmlands in the YRD studied here, which also has shallow groundwater that supports the soil water. The computed bottom flux in the studied farmlands indicates that deep percolation occurred throughout the year. The DP of winter wheat was low, and the DP of summer crops was high but far lower than that for deep groundwater or arid zones.

Although the YRD is a shallow groundwater region, CR does not contribute to agricultural water use in the area due to large interannual fluctuation of groundwater and soil salinization. If deep percolation and surface runoff were ignored in the calculation of irrigation volume in the YRD, the whole rotation season would lead to a loss of at least 5.23–9.10% of the irrigation volume. In fact, given the low effectiveness of irrigation water, this figure could be higher.

### 3.2. Saltwater Stress and Crop Coefficient

Compared with other studies on rainfed irrigation, local K_C_ values in the YRD are considered to be generally small [36,37]. On one hand, crop growth is affected by salt stress in the YRD, which in turn limits CWU. On the other hand, the high annual evapotranspiration reduces the effectiveness of water supply. This study confirmed that under the spring flood and summer rainfed irrigation scheme in the YRD, K_C_ was drastically limited. Using K_C-adj_ to guide agricultural production can cause high water waste, defeating the effort of improving water use efficiency and water conservation.

The results of the study show that CWU in the YRD was driven not only by water stress but also by salt stress. Thus, the local K_C_ values were lower than those reported in most other studies. It is therefore important to include salt as a key measure to reduce water use and improve water use efficiency in the YRD study area. 

While the combined effect of salt and water stress on WUE was smallest for soybean (4.37), it was largely similar for maize (8.70) and sorghum (9.00). The results suggest that there was no significant difference in the way C4 crops used water under the same rainfed conditions in the YRD study area as also reported in other studies [38]. In the arid Loess Plateau region, the WUE of spring maize under the same rainfed irrigation has been reported to be 27.0 kg ha^−1^ mm^−1^ [39]. In the North China plain, WUE of winter wheat in the range 17.7–20.3 kg ha^−1^ mm^−1^ has been documented [40]. Rainfed or water-deficit cultivation cannot cause significant reduction in WUE. Thus, the significant gap detected in this study was mainly due to the high salt stress in the YRD saline coastal farmland. In a salt stress experiment by saltwater irrigation, it was noted that high salt stress significantly reduces crop WUE [41]. In this study too, the combined effect of irrigation and salt stress on crop WUE in YRD was significantly low. Therefore, improving local WUE is key to saving agricultural water use in the region.

### 3.3. Suggested Cropland Management 

From the threat posed by salt stress on CWU at different crop growth stages in the YRD study area, there is a need to pay close attention to soil management measures for the period starting from sowing to overwintering of wheat in order to reduce soil salt and hence salt stress at seedling stage. As soybean can be severely affected by multiple stress factors over the period of growth, focus should be put on the selection of salt-tolerant varieties. Soil salt should be controlled for sorghum and maize particularly at the maturity stage in order to enhance grain formation, quality, and yield.

The results of the study show that the flowering stage of soybean is the most critical period for water shortage in saline coastal farmlands. The period before overwintering of wheat is also a key water shortage period. Water shortage during this period affected wheat seedling survival in the winter period, tiller at seedling stage, and grain formation at flowering stage. The maturity stage is the most critical period of water shortage for maize; for sorghum, it is the silking and grain-filling stages. For agricultural practices in the YRD study area, the focus should be on soil water conservation before overwintering of wheat. It is also necessary to winter irrigate to ensure water supply at the seedling stage of wheat. In a wheat–maize rotation, the change in soil moisture at maturity should be monitored for maize, and additional irrigation is usually required at this stage. In a wheat–sorghum rotation, irrigation should be done at the start of silking and grain-filling. In a wheat–soybean rotation, the focus should be on the change in soil moisture at the start of flowering.

Our findings offer valuable insights for improving irrigation practices at both the government and farmer levels. Given the current shortage of irrigation water in coastal saline farmlands, we recommend that the government allocate irrigation quotas based on a detailed analysis of the planting systems used by different agricultural units, the existing soil water retention and salinity in farmland, and the WUE of different crop varieties. These factors all impact the amount of irrigation needed. We also suggest that farmers adopt a collaborative “irrigation management–planting system” approach to farmland management. This entails selecting drought- or salt-resistant crop types and planting systems based on a thorough understanding of the irrigation conditions and water availability in their farmland. Additionally, farmers should carefully plan irrigation methods and soil salt management strategies, taking into account the specific planting systems used, to optimize the use of irrigation water in coastal farmland.

Overall, spring flood irrigation and summer rainfed cultivation in the study period did not meet the water needs of the current crop rotation systems, thus requiring irrigation during specific periods of growth. In terms of WUE, rotation of sorghum and maize C4 plants with high WUE are suggested for the extreme local conditions in the YRD study area.

## 4. Materials and Methods

### 4.1. Site Description

**Figure 5:**
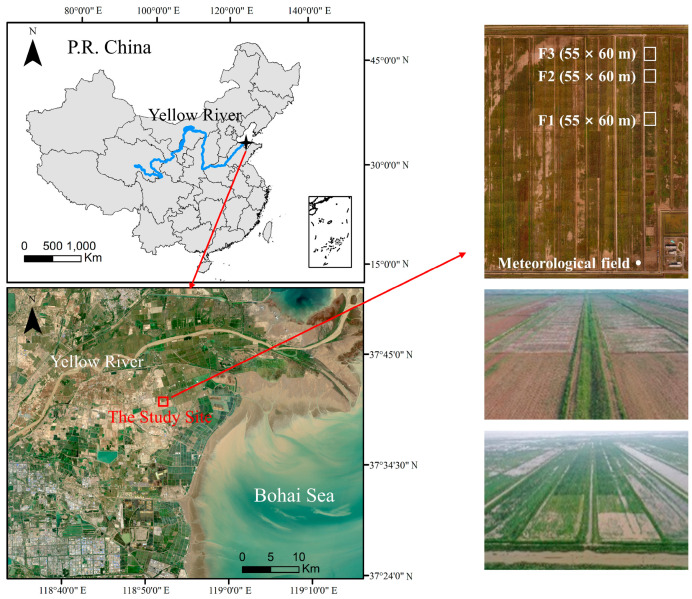
Plots depicting the location of the study site and the experimental plots. Note that the white boxes in the upper right figure represents the area of each experimental plot; F1, F2, and F3 denote three plots with wheat–maize, wheat–soybean, and wheat–sorghum rotation, respectively.

The study site was at 37°40′24″ N, 118°54′43″ E in the YRD Research Center of the Institute of Geographic Sciences and Natural Resources Research, Chinese Academy of Sciences. This is located in Kenli District in Shandong Province, China (Figure 5). In the YRD, there is considerable land–ocean interaction (seawater intrusion and groundwater recharge to the ocean) that causes large fluctuation in the water table depth. Based on the meteorological observations in the research center, the average annual temperature in the area was 13.66 °C, and the average precipitation was 581.41 mm over the past decade. The period from June to September accounts for 61.61% of the annual precipitation. The annual average evaporation is 1800 mm, which is much higher than the precipitation and a major reason for salt accumulation in the surface soil. The altitude of the study site is 0–1 m above mean sea level, and soil texture is mainly silty loam. The salt content is 3.29‰, pH 8.42, organic matter 13.28 g kg^−1^, concentration of Na^+^ 90.0 mg kg^−1^, and available nitrogen, phosphorus, and potassium of 60.64, 6.15, and 251.67 mg kg^−1^, respectively. The main crop rotation systems are wheat–maize (74.9%), wheat–rice (16.5%), wheat–soybean (7.8%), and wheat–sorghum (0.42%).

### 4.2. Experimental Design

The experiment started in October 2019 with three rotation systems set up: wheat (cv. “Xiaoyan No. 1”) and maize (cv. “Jinboshi 509”), wheat and sorghum (cv. “Kang NO. 4”), and wheat and soybean (cv. “Luhuang No. 1”). The area of each experimental plot was 55 × 60 m. Winter wheat was planted on 10 October 2019; the field was ploughed and leveled before sowing. Top-dressing and flood irrigation (300 mm) were done at the regreening stage (18 March 2020). Winter wheat matured and was harvested on 15 June 2020, i.e., after a growth period of 250 days. The summer crops maize, soybean, and sorghum were planted after wheat harvest and further field ploughing. Summer crops were top-dressed on 25 August 2020 in the rainfed condition and harvested on 15 October 2020 after another growth period of 123 days. Superphosphate (N: 0%) and nitrophosphate (N: 18%) formed the base fertilizer and urea (N: 46%) the top-dressing fertilizer in the wheat field. Nitrogen was applied in the wheat field at the rate of 180 kg N ha^−1^. In the summer field, the base fertilizer was compound fertilizer (15:15:15), and the top-dressing fertilizer was urea (N: 46%). A total of 12 soil sampling campaigns (17 March 2020, 31 March 2020, 15 April 2020, 4 May 2020, 1 June 2020, 15 June 2020, 24 July 2020, 9 August 2020, 27 August 2020, 11 September 2020, 1 October 2020, and 15 October 2020) were collected during the whole rotation period. From each plot, soil samples from depths 0–20 cm, 20–40 cm, and 40–60 cm were collected, and three replicate samples were obtained.

#### Data Collection

(a) Measurements: For every heavy precipitation and surface ponding event, the water level difference method was used to estimate surface runoff (mm) on the farmland, and the pipette method (GB7845-87) was used for soil particle size analysis. The gravimetric soil water contents in the 0–20 cm, 20–40 cm, and 40–60 cm soil layers were measured using the standard drying approach. The volumetric soil water content was calculated in combination with soil bulk density. The crop yield (kg ha^−1^) was measured at two stages of maturity.
(b) Meteorological data: Meteorological data (precipitation, wind speed, radiation, humidity, sunlight hours, etc.) were measured automatically in the nearby meteorological field every 10 min. The recorded data (Figure S1) were used to calculate the daily reference evapotranspiration (ET_O_) (mm day^−1^) using the FAO-56 Penman–Monteith equation [42]:

(1) $${\mathrm{ET}}_{O}=\frac{0.408\Delta \left(R_{n}-G\right)+\gamma \frac{900}{T+273}u_{2}\left(e_{s}-e_{a}\right)}{\Delta +\gamma \left(1+0.34u_{2}\right)}$$

where ET_O_ is the daily reference evapotranspiration [mm d^−1^], $\left(R_{n}-G\right)$ is the net balance of energy available at the crop surface [MJ m^2^ d^−1^], T is the mean daily air temperature [°C], $u_{2}$ is the wind speed at the height of 2 m [m s^−1^], e_s_ is the saturated vapor pressure [kPa], e_a_ is the actual vapor pressure, ∆ is the slope of saturated water vapor pressure curve, and γ is the psychrometric constant [kPa °C^−1^].

### 4.3. Soil Water Balance Parameterization

We used the soil water balance equation to calculate actual CWU from observed evapotranspiration (ET_C-obs_) [5,43]:

(2) $${\mathrm{ET}}_{C-\mathrm{obs}}=I+P-F-\mathrm{DP}+\mathrm{CR}\pm \Delta W$$

where ET_C-obs_ is the observed crop evapotranspiration [mm], I is the irrigation [mm], P is the precipitation [mm], DP is the deep percolation [mm], CR is the capillary rise [mm], and ΔW is the change in soil water storage [mm].

With the soil water balance method, studies ignore DP [5,8]. Here DP was obtained for each crop growth stage using the HYDRUS-1D model (see next section), which is key for accurate evaluation of ET_C_.

### 4.4. Model Simulation

#### 4.4.1. Model Equations

The HYDRUS-1D model was used to simulate soil water flow and storage. Since soil water movement and water exchange between groundwater and the soil layer mainly occurred in the vertical direction, it was not necessary to use the multi-dimensional (e.g., HYDRUS-2D/3D) model in the simulation. Thus, the flow of water in the soil was described by the 1-D Richards equation as

(3) $$\frac{\partial \theta }{\partial t}=\frac{\partial }{\partial Z}\left[K\left(h\right)\left(\frac{\partial h}{\partial Z}+1\right)\right]-S$$

where t is time [d], θ is the volumetric water content [cm^3^ cm^−3^], h is the water pressure head [cm], K(h) is the unsaturated hydraulic conductivity function [cm d^−1^], Z is the vertical spatial coordinate [cm], and S is a sink term [cm d^−1^], which is evapotranspiration [cm d^−1^] in this study.

We used the K-h and θ-h relations of the van Genuchten–Mualem equation to describe the hydraulic parameters of the experimental plots [44] as

(4) $$\theta \left(h\right)=\left\{\begin{array}{c} \theta_{r}+\frac{\theta_{s}-\theta_{r}}{{\left(l+|h\alpha {|}^{n}\right)}^{m}}(h<0)\\ \theta_{s}\left(h⩾0\right) \end{array}\right.$$

(5) $$K\left(h\right)=K_{s}S_{e}^{1}{\left[l-{\left(l-S_{e}^{\frac{l}{m}}\right)}^{m}\right]}^{2}$$

(6) $$S_{e}=\frac{\theta -\theta_{r}}{\theta_{s}-\theta_{r}}$$

where *θ_r_* and *θ_s_* are the residual and saturated volumetric water contents, respectively [cm cm^−3^]; m and n are the fitting parameters of soil water characteristic curve; *m* = 1 − (1/*n*), *n* > 1; *K*_S_ is the saturated hydraulic conductivity [cm d^−1^]; *l* is the reciprocal value of air entry suction [cm^−1^], which is generally taken as 0.5; and *S*_e_ is the relative saturation.

#### 4.4.2. Boundary Conditions

Because the effect of water table on the simulated soil layer was not negligible, the atmospheric boundary with the surface layer (including rainfall, evapotranspiration, and irrigation) and deep drainage boundary were selected as the upper and lower boundary conditions, and irrigation assumed uniform rainfall.

Since available water for root uptake of wheat, maize, soybean, and sorghum is mainly within 60 cm of the soil surface [45], the 60 cm depth of soil layer was used in the study. Other studies, such as [46], used similar shallow depths for simulations. The spatial discretization was 1 cm, and observation points were added at soil depths of 20, 40, and 60 cm. Winter wheat had limited influence on soil water transport because of its low water use during overwintering to regreening stage, less precipitation during winter, and a deep and stable groundwater during the growth period. Thus, the regreening to maturity stage of winter wheat (from 17 March 2020 to 15 June 2020) and the seedling stage to maturity stage (from 24 July 2020 to 15 October 2020) of summer crops were simulated, all using a time step of 1 day.

#### 4.4.3. Model Calibration and Evaluation

The soil profile was divided into two layers: 0–40 and 40–60 cm. The physical properties of each of the soil layers were considered to be uniform. The measured soil water states were used to set the initial conditions, and data from the simulated preceding stage were used to calibrate; the remaining stage was used to validate the hydraulic parameters of θ_r_, θ_s_, K_S_, α, and n. For wheat, the field-measured data for 17 March to 15 April 2020 were used for calibration, and those for 4 May to 15 June 2020 were used for validation. For summer crops, the field-measured data for 24 July to 27 August 2020 were used for calibration, and those for 11 September to 15 October 2020 were used for validation. The calibrated soil hydraulic parameters are shown in Table S2.

Based on the simulation results of HYDRUS-1D, three measures of goodness of fit were used to evaluate the model performance for the calibration and validation stages: the coefficient of determination (*R*^2^), Nash–Sutcliffe efficiency coefficient (NSE), and root mean square error (RMSE). The NSE is widely used to assess the performance of hydrological models. These three measures were used to assess the correlation and difference between the observed and simulated values for the calibration and validation stages, using the equations

(7) $$R^{2}={\left[\frac{\sum_{i=1}^{n}\left(P_{i}-\bar{P_{i}}\right)\left(O_{i}-\bar{O_{i}}\right)}{\sqrt{\sum_{i=1}^{n}{\left(P_{i}-\bar{P_{i}}\right)}^{2}{\sum_{i=1}^{n}\left(O_{i}-\bar{O_{i}}\right)}^{2}}}\right]}^{2}$$

(8) $$\mathrm{NSE}=1-\frac{{\sum_{i=1}^{n}\left(O_{i}-P_{i}\right)}^{2}}{{\sum_{i=1}^{n}\left(O_{i}-\bar{O_{i}}\right)}^{2}}$$

(9) $$\mathrm{RMSE}=\sqrt{\sum_{i=1}^{n}\frac{{\left(P_{i}-O_{i}\right)}^{2}}{n}}$$

where $P_{i}$ and $O_{i}$ are the daily predicted and observed values, respectively; $\bar{P_{i}}$ and $\bar{O_{i}}$ are the averages of the predicted and observed values, respectively; and n is the number of available observations.

### 4.5. Crop Coefficient

The crop coefficient (K_C_) is the ratio of ET_C_ and reference evapotranspiration (ET_O_), yielding the difference of water required by each crop at a certain growth stage. From FAO-56, the crop growing season was divided into four stages: initial (planting to 10% ground cover), crop development (from 10% ground cover to effective full cover), mid-season (from effective full cover to start of maturity), and late season (from start of maturity to harvest or full sensitivity). The number of days of each growth stage and the recommended average K_C_ value of each crop given by FAO (K_C-FAO_) are shown in Table S3. However, since the K_C-FAO_ values were for standard conditions where there were diseases and pests and with optimal soil, water, and fertilizer conditions and agricultural practices, the K_C-mid_ and K_C-end_ values (K_C_ values of mid-season and late season) were modified based on the meteorological conditions of experimental plots [42]. The modification was as follows:

(10) $$\left.K_{C-\mathrm{adj}}=K_{C-FA0}+\left[0.04\left(u_{2}-2\right)-0.004\left(RH_{\min\;}-45\right)\right)\right]{\left[\begin{array}{c} h\\ -\\ 3 \end{array}\right]}^{0.3}$$

where $K_{C-\mathrm{adj}}$ is the modified K_C_ value, $u_{2}$ is the wind speed at the height of 2 m [m s^−1^], $\;RH_{\min}$ is the average of daily minimum relative humidity [%], and h is the average plant height for a growth stage [m].

Due to the effects of salt and water stress on agricultural practices, the observed K_C_ values (K_C-obs_) of the four crops in the YRD were compared with K_C_ values under the ideal condition in order to quantify environmental stress. K_C-obs_ was calculated as the ratio of ET_C-obs_ and ET_O_, and the stress coefficient was introduced to quantify the effect of salt and water stress on CWU as follows [47]:

(11) $$K_{C-\mathrm{obs}}={\mathrm{ET}}_{C-\mathrm{obs}}/{\mathrm{ET}}_{O}$$

(12) $$Stress\;coefficient=K_{C}/K_{C-\mathrm{adj}}$$

Obviously, K_C_ values changed with different growth stages. Therefore, a crop growth curve was used to describe the change with different growth stages, expressed as [48]:

(13) $$K_{C}\left\{\begin{array}{cc} \;\;\;K_{C-\mathrm{ini}}, & t_{1}\leq t<t_{2}\\ K_{C-\mathrm{ini}}+\frac{\left(K_{C-\mathrm{mid}}-K_{C-\mathrm{ini}}\right)}{\left(t_{3}-t_{2}\right)}\left(t-t_{2}\right),\; & \;\;\;t_{2}\leq t<t_{3}\\ \;\;\;K_{C-\mathrm{mid}}, & t_{3}\leq t<t_{4}\\ {\;K}_{C-\mathrm{end}}-\frac{\left(K_{C-\mathrm{mid}}-K_{C-\mathrm{end}}\right)}{\left(t_{5}-t_{4}\right)}\left(t-t_{4}\right), & \;\;\;t_{4}\leq t\leq t_{5} \end{array}\right.$$

where $K_{C-\mathrm{ini}}$ , $K_{C-\mathrm{mid}}$ , $\;{\mathrm{and}\;K}_{C-\mathrm{end}}$ are the crop coefficients of the initial, middle, and late periods, respectively; t is the number of growing days; t_1_, t_2_, t_3_, and t_4_ are the start days of initial stage, crop development stage, mid-season stage, and end-season stage, respectively; and t_5_ is the end day of the end-season stage.

In addition to measuring CWU, water use efficiency (WUE) was calculated in terms of grain yield (GY) to quantify the contributions of CWU to crop yield in agricultural practices, using

(14) $$\mathrm{WUE}=\mathrm{GY}/{\mathrm{ET}}_{C-\mathrm{obs}}$$

## 5. Conclusions

Over the study period, groundwater in the YRD coastal farmlands appeared to have no effect on root zone water content but, instead, gained from irrigation water through deep percolation (DP). The K_C_ values of local staple food crops were highly limited by salt stress, higher than the case for many other water-stressed conditions. Changes to crop management in the YRD should focus on selecting salt-tolerant crop varieties and soil salt regulation at specific crop growth stages for high crop yields. The water use efficiencies of the three rotation systems were observed to be similar for the wheat–sorghum rotation and wheat–maize rotation and lowest for the wheat–soybean rotation in the study area. The determination of an intensive CWU period provides a scientific basis for improving local irrigation strategies and using appropriate rotation systems in saline coastal regions.

## Figures and Tables

**Table 1:** Water and salt stress coefficients in each stage of the four investigated crops and WUE in the Yellow River Delta study area.

Crop	Growth Stage	Stress Coefficient	GY (kg ha^−1^)	WUE (kg ha^−1^ mm^−1^)
Wheat	Before overwintering	0.233	3925.0	7.50
	Regreening and heading	0.912		
	Grain-filling	0.620		
	Maturity	0.431		
Maize	Emergence and jointing	0.980	3305.0	8.70
	Flare-opening	1.651		
	Silking and grain-filling	0.688		
	Maturity	0.336		
Soybean	Branching	0.608	1455.0	4.37
	Flowering	0.714		
	Grain-filling and maturity	0.286		
Sorghum	Emergence and jointing	0.550	3195.7	9.00
	Flare-opening	1.845		
	Grain-filling and maturity	0.132		

## Data Availability

The data is contained within the manuscript and Supplementary File.

## Supplementary Materials

The following supporting information can be downloaded at: https://www.mdpi.com/2223-7747/12/10/1990/s1, Figure S1: Plots of daily meteorological data for the from October 2019 to October 2020.; Figure S2: Water budget for wheat-maize, wheat-soybean and wheat-sorghum rotations in the Yellow River Delta study area. Note that SWC refers to change in soil water content [mm]; red denotes water loss and blue water gain. Rainfall, irrigation and surface runoff were obtained by in-site observations; Evapotranspiration was obtained by Penman–Monteith equation based on climate data; Bottom water flux was obtained by the simulations from HYDUS-1D model; SWC was then calculated by water balance equation.; Table S1: Goodness-of-fit test indicators of the HYDRUS-1D model calibration and validation for the Yellow River Delta study area.; Table S2: Calibrated soil hydraulic parameters of van-Genuchten equation in HYDRUS-1D; Table S3: The FAO-56 recommended average crop coefficient (K_C-FAO_) and length of crop development stage (LCS) for winter wheat, and summer maize, soybean and sorghum in the Yellow River Delta study area.

## References

[1] Brouns K., Verhoeven J.T.A., Hefting M.M. (2014). The effects of salinization on aerobic and anaerobic decomposition and mineralization in peat meadows: The roles of peat type and land use. J. Environ. Manag..

[2] Tully K.L., Weissman D., Wyner W.J., Miller J., Jordan T. (2019). Soils in transition: Saltwater intrusion alters soil chemistry in agricultural fields. Biogeochemistry.

[3] Tosi L., Da Lio C., Bergamasco A., Cosma M., Cavallina C., Fasson A., Viezzoli A., Zaggia L., Donnici S. (2022). Sensitivity, Hazard, and Vulnerability of Farmlands to Saltwater Intrusion in Low-Lying Coastal Areas of Venice, Italy. Water.

[4] Li R., Chai S.X., Chai Y.W., Li Y.W., Lan X.M., Ma J.T., Cheng H.B., Chang L. (2021). Mulching optimizes water consumption characteristics and improves crop water productivity on the semi-arid Loess Plateau of China. Agric. Water Manag..

[5] Jafari M., Kamali H., Keshavarz A., Momeni A. (2021). Estimation of evapotranspiration and crop coefficient of drip-irrigated orange trees under a semi-arid climate. Agric. Water Manag..

[6] Nicolas E., Alarcon J.J., Mounzer O., Pedrero F., Nortes P.A., Alcobendas R., Romero-Trigueros C., Bayona J.M., Maestre-Valero J.F. (2016). Long-term physiological and agronomic responses of mandarin trees to irrigation with saline reclaimed water. Agric. Water Manag..

[7] Wang T.Y., Xu Z.H., Pang G.B. (2019). Effects of Irrigating with Brackish Water on Soil Moisture, Soil Salinity, and the Agronomic Response of Winter Wheat in the Yellow River Delta. Sustainability.

[8] Xie T., Liu X.H., Sun T. (2011). The effects of groundwater table and flood irrigation strategies on soil water and salt dynamics and reed water use in the Yellow River Delta, China. Ecol. Model..

[9] Anapalli S.S., Ahuja L.R., Gowda P.H., Ma L.W., Marek G., Evett S.R., Howell T.A. (2016). Simulation of crop evapotranspiration and crop coefficients with data in weighing lysimeters. Agric. Water Manag..

[10] Parish A.L., Kendall A.D., Thompson A.M., Stenjem R.S., Hyndman D.W. (2019). Cellulosic biofuel crops alter evapotranspiration and drainage fluxes: Direct quantification using automated equilibrium tension lysimeters. Glob. Chang. Biol. Bioenergy.

[11] Angus D.E., Watts P.J. (1984). Evapotranspiration—How Good Is the Bowen-Ratio Method. Agric. Water Manag..

[12] Sousa D.D., Fernandes T.F.S., Tavares L.B., Farias V.D.D., de Lima M.J.A., Nunes H.G.G.C., Costa D.L.P., Ortega-Farias S., Souza P.J.D.P. (2021). Estimation of evapotranspiration and single and dual crop coefficients of acai palm in the Eastern Amazon (Brazil) using the Bowen ratio system. Irrig. Sci..

[13] Ding J., Li S.E., Wang H.S., Wang C.Y., Zhang Y.X., Yang D.N. (2021). Estimation of Evapotranspiration and Crop Coefficient of Chinese Cabbage Using Eddy Covariance in Northwest China. Water.

[14] Li S., Kang S.H., Li F.S., Zhang L. (2008). Evapotranspiration and crop coefficient of spring maize with plastic mulch using eddy covariance in northwest China. Agric. Water Manag..

[15] Nagler P.L., Scott R.L., Westenburg C., Cleverly J.R., Glenn E.P., Huete A.R. (2005). Evapotranspiration on western US rivers estimated using the Enhanced Vegetation Index from MODIS and data from eddy covariance and Bowen ratio flux towers. Remote Sens. Environ..

[16] Cha M.X., Li M.M., Wang X.Q. (2020). Estimation of Seasonal Evapotranspiration for Crops in Arid Regions Using Multisource Remote Sensing Images. Remote Sens..

[17] Pereira L.S., Allen R.G., Smith M., Raes D. (2015). Crop evapotranspiration estimation with FAO56: Past and future. Agric. Water Manag..

[18] Shao G.M., Han W.T., Zhang H.H., Liu S.Y., Wang Y., Zhang L.Y., Cui X. (2021). Mapping maize crop coefficient Kc using random forest algorithm based on leaf area index and UAV-based multispectral vegetation indices. Agric. Water Manag..

[19] Zhang Q., Wang W., Yang F. (2019). Drought-stress modified crop coefficients for estimation of evapotranspiration of spring wheat in the southwest Loess Plateau of China. J. Soil Water Conserv..

[20] Payero J.O., Irmak S. (2013). Daily energy fluxes, evapotranspiration and crop coefficient of soybean. Agric. Water Manag..

[21] Chen H.Y., Yang C., Ren A.Y., Guo K., Feng X.H., Li J.S., Liu X.J., Sun H.Y., Wang J.L. (2019). The Evapotranspiration of Tamarix and Its Response to Environmental Factors in Coastal Saline Land of China. Water.

[22] Katerji N., Mastrorilli M., Lahmar F. (2011). FAO-56 methodology for the stress coefficient evaluation under saline environment conditions: Validation on potato and broad bean crops. Agric. Water Manag..

[23] Han M., Zhao C.Y., Simunek J., Feng G. (2015). Evaluating the impact of groundwater on cotton growth and root zone water balance using Hydrus-1D coupled with a crop growth model. Agric. Water Manag..

[24] Xiao X., Xu X., Ren D.Y., Huang Q.Z., Huang G.H. (2021). Modeling the behavior of shallow groundwater system in sustaining arid agroecosystems with fragmented land use. Agric. Water Manag..

[25] Lena B.P., Bondesan L., Pinheiro E.A.R., Ortiz B.V., Morata G.T., Kumar H. (2022). Determination of irrigation scheduling thresholds based on HYDRUS-1D simulations of field capacity for multilayered agronomic soils in Alabama, USA. Agric. Water Manag..

[26] Tafteh A., Sepaskhah A.R. (2012). Application of HYDRUS-1D model for simulating water and nitrate leaching from continuous and alternate furrow irrigated rapeseed and maize fields. Agric. Water Manag..

[27] Davis K.F., Rulli M.C., Seveso A., D’Odorico P. (2017). Increased food production and reduced water use through optimized crop distribution. Nat. Geosci..

[28] Jiang Y., Xu X., Huang Q.Z., Huo Z.L., Huang G.H. (2016). Optimizing regional irrigation water use by integrating a two-level optimization model and an agro-hydrological model. Agric. Water Manag..

[29] Xu X., Sun C., Qu Z.Y., Huang Q.Z., Ramos T.B., Huang G.H. (2015). Groundwater Recharge and Capillary Rise in Irrigated Areas of the Upper Yellow River Basin Assessed by an Agro-Hydrological Model. Irrig. Drain..

[30] Tan X.Z., Shao D.G., Liu H.H. (2014). Simulating soil water regime in lowland paddy fields under different water managements using HYDRUS-1D. Agric. Water Manag..

[31] Babajimopoulos C., Panoras A., Georgoussis H., Arampatzis G., Hatzigiannakis E., Papamichail D. (2007). Contribution to irrigation from shallow water table under field conditions. Agric. Water Manag..

[32] Petheram C., Dawes W., Grayson R., Bradford A., Walker G. (2003). A sub-grid representation of groundwater discharge using a one-dimensional groundwater model. Hydrol. Process..

[33] Dash C.J., Sarangi A., Singh D.K., Adhikary P.P. (2019). Numerical simulation to assess potential groundwater recharge and net groundwater use in a semi-arid region. Environ. Monit. Assess..

[34] Li X.D., Zhao Y., Xiao W.H., Yang M.Z., Shen Y.J., Min L.L. (2017). Soil moisture dynamics and implications for irrigation of farmland with a deep groundwater table. Agric. Water Manag..

[35] Cameira M.R., Fernando R.M., Pereira L.S. (2003). Monitoring water and NO3-N in irrigated maize fields in the Sorraia Watershed, Portugal. Agric. Water Manag..

[36] Djaman K., Irmak S. (2013). Actual Crop Evapotranspiration and Alfalfa- and Grass-Reference Crop Coefficients of Maize under Full and Limited Irrigation and Rainfed Conditions. J. Irrig. Drain. Eng..

[37] Gao Y., Yang L.L., Shen X.J., Li X.Q., Sun J.S., Duan A.W., Wu L.S. (2014). Winter wheat with subsurface drip irrigation (SDI): Crop coefficients, water-use estimates, and effects of SDI on grain yield and water use efficiency. Agric. Water Manag..

[38] Roby M.C., Fernandez M.G.S., Heaton E.A., Miguez F.E., VanLoocke A. (2017). Biomass sorghum and maize have similar water-use-efficiency under non-drought conditions in the rain-fed Midwest US. Agric. For. Meteorol..

[39] Liu Y., Li S.Q., Chen F., Yang S.J., Chen X.P. (2010). Soil water dynamics and water use efficiency in spring maize (*Zea mays* L.) fields subjected to different water management practices on the Loess Plateau, China. Agric. Water Manag..

[40] Guan D.H., Zhang Y.S., Al-Kaisi M.M., Wang Q.Y., Zhang M.C., Li Z.H. (2015). Tillage practices effect on root distribution and water use efficiency of winter wheat under rain-fed condition in the North China Plain. Soil Tillage Res..

[41] Wang Q.M., Huo Z.L., Zhang L.D., Wang J.H., Zhao Y. (2016). Impact of saline water irrigation on water use efficiency and soil salt accumulation for spring maize in arid regions of China. Agric. Water Manag..

[42] Allen R.G., Pereira L.S., Raes D., Smith M. (1998). Crop Evapotranspiration. Guidelines for Computing Crop Evapotranspiration.

[43] Sepaskhah A.R., Andam M. (2001). Crop coefficient of sesame in a semi-arid region of IR Iran. Agric. Water Manag..

[44] Van Genuchten M.T. (1980). A Closed-Form Equation for Predicting the Hydraulic Conductivity of Unsaturated Soils. Soil Sci. Soc. Am. J..

[45] Hou C.L., Tian D.L., Xu B., Ren J., Hao L., Chen N., Li X.Y. (2021). Use of the stable oxygen isotope method to evaluate the difference in water consumption and utilization strategy between alfalfa and maize fields in an arid shallow groundwater area. Agric. Water Manag..

[46] Er-Raki S., Ezzahar J., Merlin O., Amazirh A., Hssaine B.A., Kharrou M.H., Khabba S., Chehbouni A. (2021). Performance of the HYDRUS-1D model for water balance components assessment of irrigated winter wheat under different water managements in semi-arid region of Morocco. Agric. Water Manag..

[47] Doorenbos J., Pruitt W.O. (1977). Crop Water Requirements.

[48] Juan C.H., Shih S.F. (1997). A lysimeter system for evapotranspiration estimation for wetland vegetation. Soil Crop Sci. Soc. Fla. Proc..

