# Supplementary material for: Irrigation Optimization via Crop Water Use in Saline Coastal Areas—A Field Data Analysis in China’s Yellow River Delta
Source: Plants (Basel). 2023 May 15;12(10):1990. doi: 10.3390/plants12101990 (PMC10223565; doi:10.3390/plants12101990)
Supplement: Supplementary file 1 [file plants-12-01990-s001.zip › plants-2381289-supplementary.pdf]

plants
MDPI

Supplementary materials

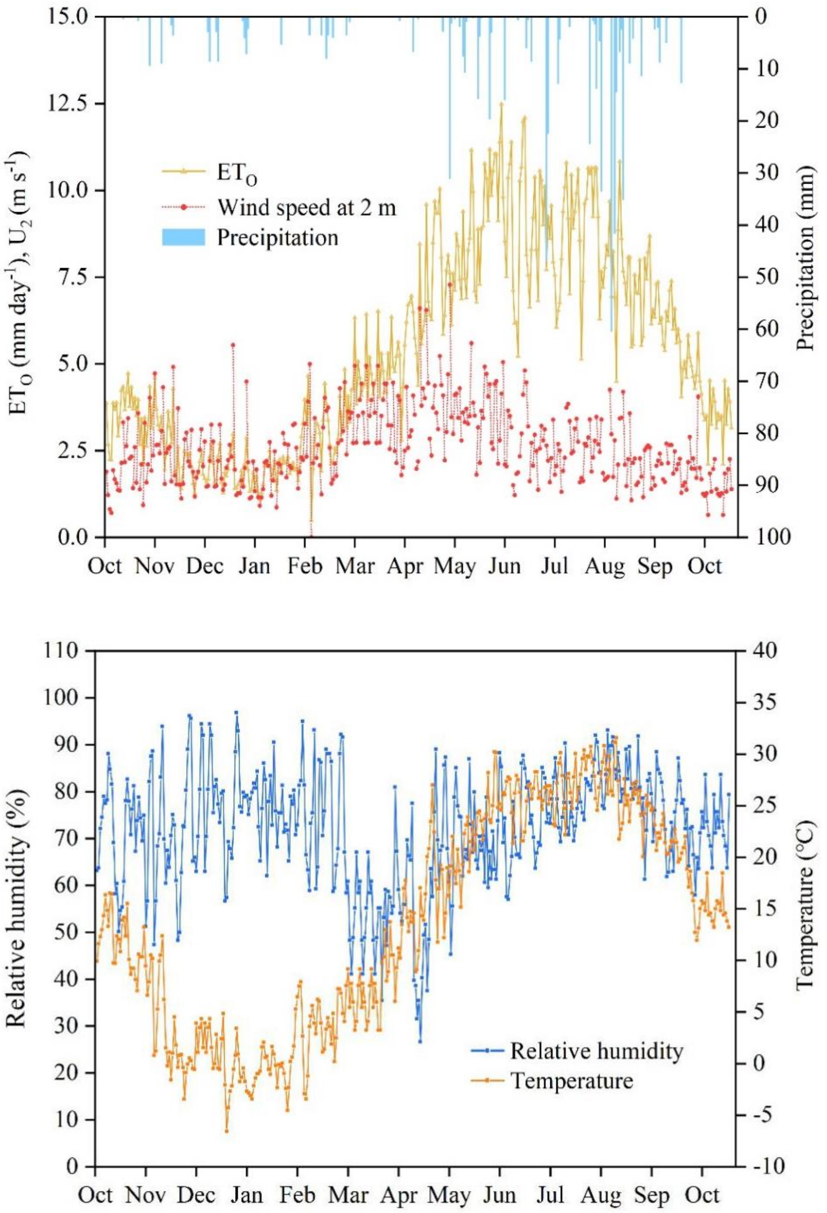

Figure S1. Plots of daily meteorological data for the from October 2019 to October 2020.

Table S1. Goodness-of-fit test indicators of the HYDRUS-1D model calibration and validation for the Yellow River Delta study area.

**Period**	**Crop**	**R^2**	**RMSE ($cm^3\ cm^{-3}$)**	**NSE**
Calibration	Wheat	0.98	0.012	0.98
	Soybean	0.99	0.005	0.99
	Maize	0.88	0.022	0.82
	Sorghum	0.97	0.014	0.97
Validation	Wheat	0.60	0.052	0.46
	Soybean	0.99	0.012	0.98
	Maize	0.98	0.016	0.96
	Sorghum	0.92	0.026	0.92

R^2, RMSE and NSE are the coefficient of determination, Nash–Sutcliffe efficiency coefficient and root mean square error, respectively.

Table S2. Calibrated soil hydraulic parameters of van-Genuchten equation in HYDRUS-1D.

**Crops**	Soil depths	Hydraulic parameters in the van Genuchten model				
		θ_r ($cm^3\ cm^{-3}$)	θ_s ($cm^3\ cm^{-3}$)	α (cm^{-1})	n	K_s ($cm\ d^{-1}$)
**Wheat**	0-40 cm	0.025	0.517	0.007	1.35	59.3
	40-60 cm	0.040	0.551	0.006	1.49	37.7
Maize	0-40 cm	0.102	0.408	0.093	2.26	31.8
	40-60 cm	0.107	0.408	0.105	1.98	73.2
Soybean	0-40cm	0.106	0.378	0.074	1.44	24.9
	40-60 cm	0.106	0.419	0.073	2.37	70.5
Sorghum	0-40 cm	0.109	0.345	0.028	1.24	24.1
	40-60 cm	0.106	0.477	0.021	5.93	25.3

Table S3. The FAO-56 recommended average crop coefficient ($K_{C\text{-}FAO}$) and length of crop development stage (LCS) for winter wheat, and summer maize, soybean and sorghum in the Yellow River Delta study area.

[a] $K_{C\text{-}FAO}$ values are the crop coefficients of initial/mid-season/late-season given by FAO.

**Crop**	**$K_{C\text{-}FAO}$[a]**	**LCS (days)[b]**
Wheat	0.70/1.15/0.25	30/40/30
Maize	0.30/1.20/0.35	20/40/30
Soybean	0.50/1.15/0.50	20/60/25
Sorghum	0.30/1.20/1.05	20/40/30

[b] LCS is the number of days of initial/mid-season/late-season stages. The values of wheat, and summer maize, soybean and sorghum are respectively from Central USA, Nigeria and USA.

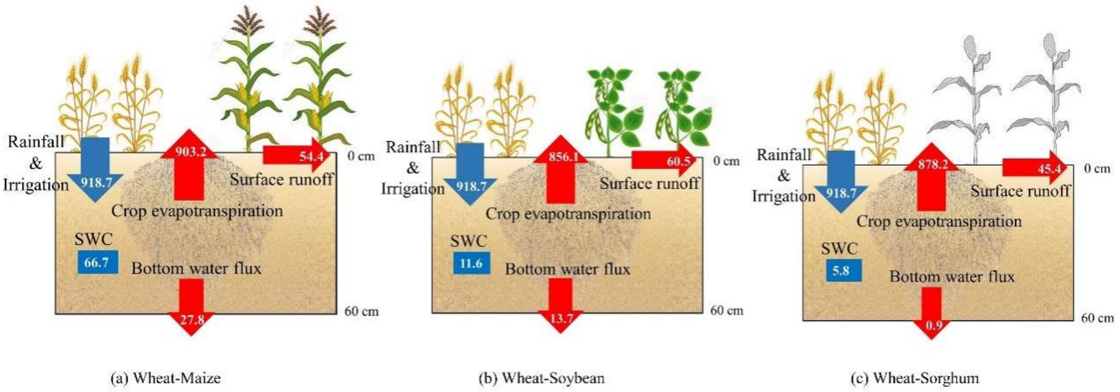

Figure S2. Water budget for wheat-maize, wheat-soybean and wheat-sorghum rotations in the Yellow River Delta study area. Note that SWC refers to change in soil water content [mm]; red denotes water loss and blue water gain. Rainfall, irrigation and surface runoff were obtained by in-site observations; Evapotranspiration was obtained by Penman–Monteith equation based on climate data; Bottom water flux was obtained by the simulations from HYDUS-1D model; SWC was then calculated by water balance equation.
